# Interdisciplinary Approach Toward Reirradiation of Cancer Patients

**DOI:** 10.7759/cureus.65750

**Published:** 2024-07-30

**Authors:** Shweta B Dahake, Ashish Uke, Anurag Luharia, Monika Luharia, Gaurav V Mishra, Chanrashekhar Mahakalkar

**Affiliations:** 1 Medical Physics, Datta Meghe Institute of Higher Education and Research, Wardha, IND; 2 Radiotherapy, Datta Meghe Institute of Higher Education and Research, Wardha, IND; 3 Medical Physics and Radiology, Datta Meghe Institute of Higher Education and Research, Wardha, IND; 4 Siddhant and Samhita, Ayurveda, Datta Meghe Institute of Higher Education and Research, Wardha, IND; 5 Radiodiagnosis, Datta Meghe Institute of Higher Education and Research, Wardha, IND; 6 General Surgery, Datta Meghe Institute of Higher Education and Research, Wardha, IND

**Keywords:** brachytherapy, sbrt, medical physics management, medical physics consult, reirradiation

## Abstract

As systemic therapies, alongside radiation, for cancer treatment continue to evolve, the radiation oncology community is facing an increasing number of reirradiation (re-RT) of tumor sites subject to recurrences. There are multiple factors associated with choosing re-RT as a treatment option for a previously irradiated site. The factors include the site of previous radiotherapy (RT), the current extent of the disease, the nature of recurrence, the technique used for previous irradiation, and the previous RT details including dose and dose fractionation. There is a persistent heterogeneity in the workflow and decision-making in cancer care centers worldwide. The current review is an attempt to dive into the practices of decision-making for re-RT, interdisciplinary attention given to the re-RT patients, and acceptable doses to the organ at risk (OAR) deduced from the understanding of previous RT and radiobiology of the tumor and sites evidence of better techniques for effective execution.

## Introduction and background

Cancer treatment includes surgery, radiotherapy (RT), and chemotherapy (CT). About two-thirds of the cancers are now getting treated via radiation therapy with or without CT. The intent of radiation can be either radical or palliative. RT has evolved from conventional treatment to image-guided RT (IGRT) in the last century. Despite the advancement of various technologies in radiation techniques, incidences of recurrences are increasing in patients who received radiation at the same site [1]. The application of the second course of RT in a recurrent volume that overlaps with that of a previously given course of radiation therapy is termed reirradiation, also re-RT. Re-RT requires extensive parameters for patient selection, such as radical or palliative, recurrent, metastatic, or second cancers concerning the previously present condition. Also, radiation treatment technology and imaging developments have provided highly accurate targeting of biologically relevant tumor volumes. Re-RT must be administered with optimum care while keeping the doses to the organ at risk (OAR) to the minimal possible values and the best tumor coverage. The review mainly focuses on the present state of re-RT scenarios and the clinical physicist's perspective with overall involvement in decision-making and treatment planning via extensive medical physics counseling [1].

## Review

Rationale

There is a significant increase in the number of patients undergoing RT at the same site as earlier, i.e., re-RT for local recurrence. Considering the number of cases of re-RT, there needs to be more discussion around the standard of practice and the outcomes of the same. This review intends to explore and navigate the available options for re-RT and attempts to put together the methodology/procedure stated by various studies for decision-making and clinical judgment.

Clinical physicist approach

**Figure 1:**
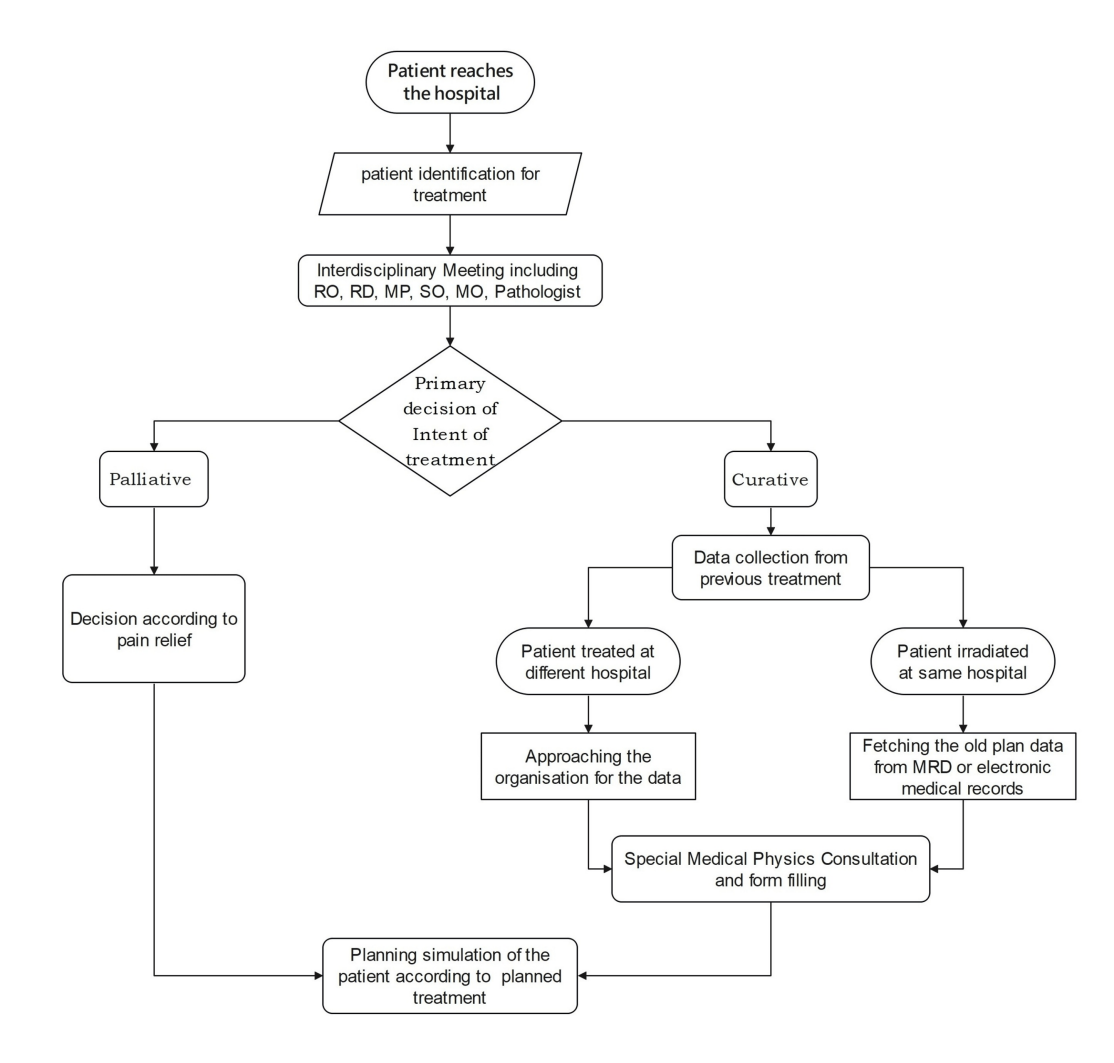
Pre-planning workflow for reradiation patient RO: Radiation oncologist; RD: radiodiagnosis; MP: medical physicist; SO: surgical oncologist; MO: medical oncologist; MRD: medical record department; CO: clinical objectives; SOP: standard of practice Image credit: Shweta Dahake

**Figure 2:**
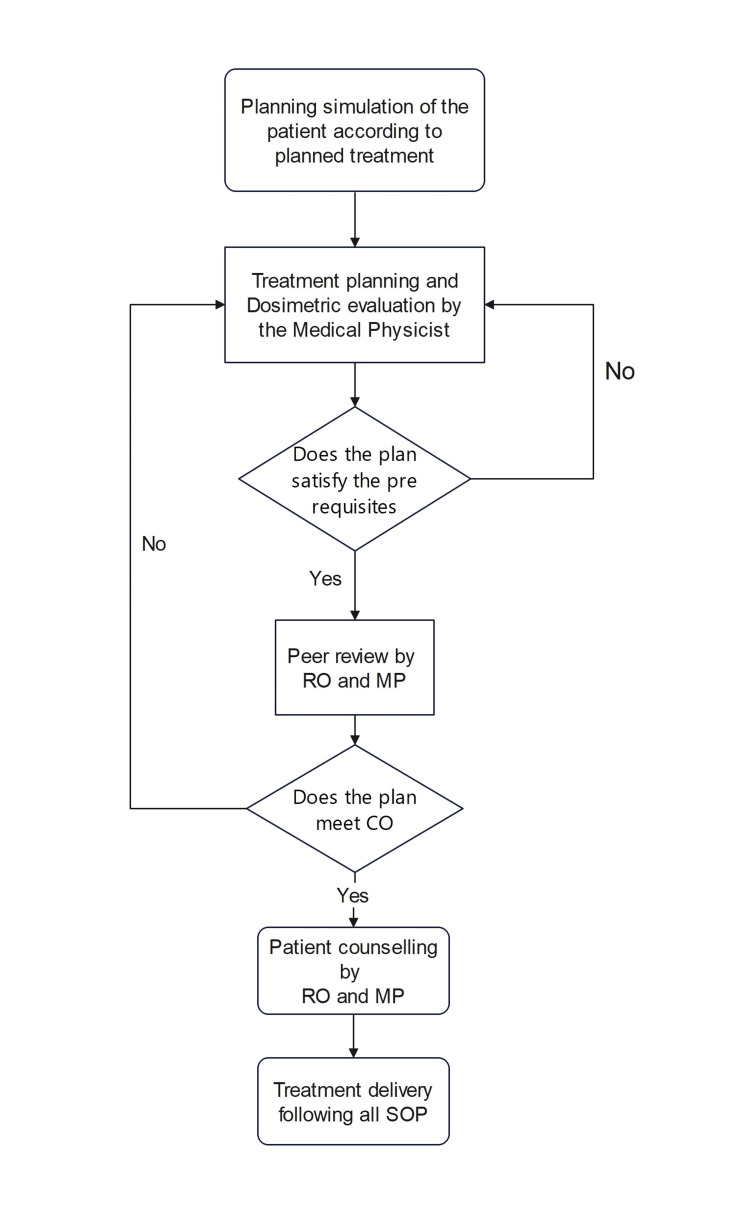
Workflow from simulation to treatment RO: Radiation oncologist; RD: radiodiagnosis; MP: medical physicist; SO: surgical oncologist; MO: medical oncologist; MRD: medical record department; CO: clinical objectives; SOP: standard of practice Image credit: Shweta Dahake

Clinical medical physicists have been integral in RT and dosimetry since the beginning. With various technological advancements in treatment planning and treatment delivery techniques, the presence becomes even more crucial in decision-making and management. A clinical physicist needs to understand the machine parameters, feasibility, and restrictions involved in dose delivery, various clinical aspects of dose distributions and planning, and quality and safety assurance customized for individual patients. While the set of skills is essential for each treatment, it becomes even more profound regarding the irradiated site or volume, whether curative or palliative intent. There is a relative scarcity of high-quality evidence to ensure homogeneous standards and best practices regarding re-RT, as there is a need to balance tumor control and the risk of severe toxicities from cumulative radiation doses to the previously irradiated tumor volume and OARs. A survey conducted in 2022 by Willmann et al. included a total of 15 MCQs focusing on various aspects of the re-RT process answered by professionals, who pointed out the heterogeneity in re-RT practices, which demands an interdisciplinary collaboration to evaluate re-RT across different tumor types, using various fractionation schemes [2]. A French survey by Ayadi et al. collected data from a group of clinical physicists and clinicians from 48 institutions across France. Out of the respondents, nearly half of the institutions discussed re-RT cases in a multidisciplinary meeting, and the prime focus was indications, previous radiation doses, and accumulated dose estimation. In contrast, most of the problems faced were regarding the decision of the cumulative equivalent dose in 2 Gy fractions (EQD2) limits for OARs. The most significant technical difficulty raised by the professionals was related to data from previous treatments and the data collection process. Also, they stated that treatment delivery accuracy, training, and clinical experience are prerequisites for sound management of re-RT [3]. As mentioned in the Medical Physicist Practice Guidelines (MPPG) 11 a, a well-maintained treatment planning report, including treatment plan parameters, should preferably be documented and archived in the hospital's Electronic Medical Record (EMR) system, which will be an integral part of the re-RT setting as recommended. The clinical medical physicist is responsible for treatment planning and replanning if needed. Hence, documentation is also a crucial part of the re-RT planning for medical physicists [4]. The medical physics management of re-RT patients needs a rigorous and efficient workflow, deduced by Paradis et al. in the form of Retreatment Special Medical Physics Consult (SMPC) at the University of Michigan. This involves identifying previously irradiated patients and data collection for the same. A formal request for re-RT SMPC followed by multiple cycles of treatment planning and analysis until finalization by physicists, followed by peer review. Also, the further process of delivery and execution involves chart rounds and one-on-one reviews of SMPC summary reports. A consult across radiation oncology departments in the United States, through the same study, showed a requirement for viewing and optimizing the biological dose with the help of software system features [5]. A parallel study regarding the generation and implementation of a practical, structured process allowing for consistent, safe RT delivery in the retreatment environment was done in 2020 by Price et al. at the Department of Radiation Oncology, Fox Chase Cancer Center, Philadelphia, Pennsylvania. This involved the Special Physics Consultation for evaluating previous treatment followed by data collection of previous treatment. After plan generation, the physicist creates a complete special physics consultation document, including assumptions made w.r.t. ratio and percent repair for the OARs. Finally, the sum of the physical dose to the endpoint of interest and EQD2, with or without repair, is recorded and sent for further processing [6]. Medical physicists specialize in skills to set up mathematical descriptions of biological and clinical problems with appropriate expertise; they will also be able to contribute to modelling new sets of guidelines/protocols in re-RT. European Societies for Radiotherapy and Oncology (ESTRO), in 2022, conducted a physics workshop in dose summation for plan optimization, evaluation, and outcome analysis for re-RT. The prevalence of heterogenous practices, poor quality data, and lack of standard approach in dose assessment still need to be solved by establishing standards of practice at the national and international levels or even at the institutional levels. Figure 1 shows the workflow for reradiation of a patient from the visit to the treatment simulation. Figure 2 shows the further process from simulation to treatment delivery.

Site-specific studies

Central Nervous System (CNS) Tumors

Though CNS tumors undergo aggressive management, including surgery, RT, and systemic therapy delivered alone or in combination, a significant proportion of patients with CNS tumors experience tumor recurrence. Radiation oncologists and medical physicists have been cautious about reirradiating brain tumors because of concerns of late toxicity when exceeding normal tissue constraints, especially radionecrosis, which can occur several months to many years following treatment [7]. Re-RT of brain tumors is attracting more interest as our understanding of the tolerance of the brain to RT evolves. The extent of surgical resection at relapse is limited by the inﬁltrative nature of these tumors and the need to avoid severe neurological deﬁcit from further surgical intervention. Re-RT is considered local therapy which independently has demonstrated acceptable median survival times of 8-11 months. The poorer prognostic factors associated in patients with reradiation are increased age, lower Karnofsky Performance Status (KPS), glioblastoma multiforme histology (GBM), frequent steroid use, short disease-free survival (DFS), and frontal lobe [8]. The various techniques for re-RT for cranial tumors are listed below.

Brachytherapy: Chatzikonstantinou et al. from University Hospital Frankfurt, Germany, shared her 20-year single institution experience for 120 patients with CT-guided interstitial high-dose rate (HDR) brachytherapy for GBM and concluded that it was an effective re-RT method for larger recurrent tumors without excessive toxicity [9]. A cumulative experience of 91 patients from 10 institutions for the GliaSite RT planning system in recurrent malignant gliomas has been investigated by Gabayan. After recurrence, each patient underwent maximal surgical debulking of their recurrent lesion and placement of an expandable balloon catheter (GliaSite) in the tumor cavity. The balloon was afterloaded with liquid 125I (Iotrex) to deliver a median dose of 60 Gy to an average depth of 1 cm with a median dose rate of 52.3 Gy/hr. The median survival for all patients, measured from the date of GliaSite placement, was 36.3 weeks with an estimated one-year survival of 31.1% showing survival benefit as compared to surgery alone [10].

Stereotactic body radiation therapy (SBRT): A small study from the Department of Surgery, in Sydney, Baltimore, and Philadelphia, by Fogh, with 147 patients of recurrent high-grade glioma treated with SBRT with a median dose of 35 Gy in 3.5 Gy fractions showed a median survival of 11 months post-re-RT [11]. A similar study by Palmer et al. from Bodine Center for Cancer Treatment, Philadelphia, for 131 patients with recurrent high-grade glioma showed a median overall survival (OS) of 10-11 months (p < 0.009) [12].

Carbon ion: The role of carbon ion RT compared with photon radiation was evaluated in an explorative hypothesis-generating a retrospective analysis by Combs with 48 patients of glioma, which showed median OS for photon therapy. Carbon ion therapy was nine and 18 months, respectively, indicating a potential benefit of carbon ions in high-grade glioma [13]. 

Head and Neck Tumors 

The standard head and neck cancer treatment includes surgery and/or RT. Despite extensive surgery and modest treatment planning, half of the patients with locally advanced head and neck cancers exhibit locoregional recurrence. These patients with secondary or recurrent cancer have a poor prognosis with a median OS of less than one year. They experience various symptoms like pain, dysphagia, bleeding, speech impairment, and respiratory distress. As surgery is considered a good salvage option, approximately half of the patients remain unresectable. CT can be considered a good option but has a low response rate and limited efficacy. The more robust and essential option, i.e., salvage re-RT, has the advantage of attaining optimal tumor control and reduced toxicities without worsening the patient's quality of life. The role of RT in re-RT settings was tried in the pre-intensity-modulated radiation therapy (IMRT) era in radiation therapy oncology group (RTOG) 9610 [14] and RTOG 9911 [15], with a two-year OS rate of 15 and 25 %, respectively, as discussed in detail below.

Pre-IMRT studies: An RTOG 9610 phase II clinical trial tried to identify acute and late side effects along with two-year survival in patients with recurrent squamous cell head and neck cancer treated with re-RT. RT was performed using standard lateral opposing, single-wedge paired, or oblique fields with a more than 1.25 MV photon and 4-25 MeV electron energy. A total of 83 patients with recurrent head and neck cancers were included in the study, which showed one- and two-year survival rates of 42.6 % and 17.3%, respectively. The final report of the study published in 2007 showed a re-RT as a practical and feasible approach with nearly acceptable toxicities [14]. A phase II clinical trial (RTOG 9911) included 105 patients receiving reradiation twice a day (1.5 Gy BID, for five days) along with concurrent CT (cisplatin and paclitaxel). The RT treatment field consists of recurrent tumors, including planning target volume (PTV) margins of 2 cm. They observed a median survival time of 12.1 months with estimated one- and two-year OS of 50.2% and 25.9%, respectively. Grade IV or worse acute toxicity was present in 28% of patients, along with eight treatment-related deaths. The trial concluded that despite higher incidents of toxicities, chemoradiotherapy (CTRT) with split-course RT exceeds CT alone in terms of OS rates [15].

IMRT: IMRT uses multiple beamlets to target irregularly shaped tumors and optimize dose escalation while sparing normal tissue. Several studies have tried to assess the effectiveness of IMRT in recurrent head and neck cancers. A meta-analysis and systematic review regarding IMRT in recurrent head neck cancer by Lee et al. included 17 studies with 1635 patients. The median dose ranges from 59.4 to 70 Gy, and the median time interval between radiation courses ranges from 14 to 41.9 months. It was found that the two-year local control (LC) rate ranged from 36% to 65%, and for the same, the OS rate ranged from 32% to 59%. Grade ≥ III late toxicities ranged from 14.2% to 57.1%, whereas grade V late toxicities ranged from 1.3% to 7.4%. The most common cause of grade V complications observed was carotid rupture, followed by soft tissue necrosis. In conclusion, re-RT with IMRT improved survival and safety compared to pre-IMRT modalities [16]. Table 1 includes the relevant IMRT studies having impactful results of IMRT.

**Table 1:** Relevant studies showing impactful results of IMRT LC: Local control; OS: overall survival; IMRT: intensity-modulated radiation therapy

Sr. No.	Study Name	Author	Number of Patients	Result
1	Reirradiation of head and neck cancers with intensity modulated radiation therapy: outcomes and analyses	Takiar et al. [17]	227	Five-year locoregional control, progression-free survival, and OS rates of 53%, 22%, and 32%
2	A nomogram to predict loco-regional control after re-irradiation for head and neck cancer	Riaz et al. [18]	257	Two-year LRC and OS were 47% and 43%
3	Refining patient selection for reirradiation of head and neck squamous carcinoma in the IMRT era: a multi-institution cohort study by the MIRI collaborative	Ward et al. [19]	412	

SBRT: SBRT has advantages with a high dose per fraction with reduced treatment time, leading to reduced overall treatment time and, hence, decreased recurrences. High dose per fraction of SBRT near vessels can lead to carotid blowout syndrome (CBOS) and can contribute to lethal toxicity. So, precautions must be taken while selecting the patients for SBRT in the real setting. The largest meta-analysis on re-RT using stereotactic body RT in managing recurrent or second primary head and neck cancers by Lee et al. included 10 studies involving 575 patients. The median target volume of SBRT re-RT was 19 to 103 cm^3^, and median SBRT re-RT doses ranged from 24 to 44 Gy (median 30 Gy) delivered with 3-6 fractions (median five fractions). Results showed that the clinical and complete responses were 61.7% and 31.3%, respectively, and the two-year LC rate was 47.3%. This study concluded that SBRT is a feasible therapy showing good responses for patients with recurrent head and neck cancers [20]. The other important studies showing similar outcomes in recurrent head and neck cancers with SBRT are listed in Table 2.

**Table 2:** Relevant studies showing impactful results of SBRT LC: Local control; OS: overall survival; SBRT: stereotactic body radiation therapy

Sr. No.	Study Name	Author	Number of Patients	Result
1	Reirradiation using robotic image-guided stereotactic radiotherapy of recurrent head and neck cancer	Yamazaki et al. [21]	107	Two-year OS rate 35%.
2	The impact of tumor volume and radiotherapy dose on outcome in previously irradiated recurrent squamous cell carcinoma of the head and neck treated with stereotactic body radiation therapy	Rwigema et al. [22]	96	One-year OS rate was 58.9, and two-year OS was 28.4%
3	Safety and efficacy of hypofractionated stereotactic body reirradiation in head and neck cancer: Long-term follow-up of a large series	Kress et al. [23]	85	Two-year Kaplan–Meier estimates of overall survival (OS) and locoregional control for patients and lesions treated with curative intent were 24% and 28%

Brachytherapy: Brachytherapy with or without salvage surgery is used as an adjuvant therapy in recurrent head and neck cancer. Characteristics of brachytherapy include conformal dose delivery while minimizing dose to adjacent noncancerous tissue, thus reducing normal tissue side effects. Brachytherapy can be delivered via low dose rate (LDR) or high dose rate (HDR) permanent implants or removable catheters. A systematic review by Rodin et al. regarding the role of brachytherapy in the treatment of recurrent head and neck cancer consisted 23 studies of patients treated with brachytherapy with or without surgery, and they concluded that it can be an effective treatment option with acceptable toxicities with a reported local regional (LR) control rate and OS at two years ranging from 27.5% to 92.5% and 18.2% to 40%, respectively [24]. Table 3 summarizes relevant studies showing similar outcomes.

**Table 3:** Relevant studies showing impactful results of brachytherapy LC: Local control; OS: overall survival

Sr. No.	Name of the Study	Author	Number of Patients	Results
1	Interstitial low-dose-rate brachytherapy as a salvage treatment for recurrent head-and-neck cancers: long-term results	Puthawala et al. [25]	220	LC at five years: 51% OS at five years: 20%
2	Reirradiation for recurrent head and neck cancer with salvage interstitial pulsed-dose-rate brachytherapy: long-term results	Strnad et al. [26]	104	LC at five years: 57%
3	High-dose-rate brachytherapy in the treatment of recurrent and residual head and neck cancer	Glatzel et al. [27]	90	Median: OS six months

*Lung Cancer* 

Despite definitive surgery or radiation for lung cancer, locoregional recurrences are a frequent type of failure. Recent roles of irradiation in lung cancer are emerging with the latest advancements in RT techniques and delivery, resulting in better outcomes. However, most studies are retrospective and do not include small-cell histology. With novel technologies like stereotactic ablative RT (SABR) and technological advances like cyber knives, protons and carbon ions have become important tools in treating patients more successfully. Curative intent with re-RT is possible with good KPS and smaller volume, but the patients who do not pass the selection criteria for re-RT are supported by palliative care. The effective re-RT techniques available are brachytherapy, IMRT, and proton beam therapy (PBT) and are listed and discussed below.

Brachytherapy: Endobronchial brachytherapy is used for previously irradiated lung cancer with mixed histologies, but the minority histology is small cell lung cancer. The first reported endobronchial brachytherapy by Mendiondo from the University of Kentucky Medical Center and Veterans Administration Hospital, Lexington, KY, using an endotracheal tube with three sesium-137 sources implanted for 18 hours, delivered a dose of 3311 rad at 0.5 cm and 1293 rad at 1 cm and showed complete remission on repeated bronchoscopy [28]. Another study included 41 patients treated with HDR endo-luminal brachytherapy with a median dose of 15 Gy and a median interval between primary external beam radiation therapy (EBRT) and re-RT of nine months. The median OS observed in the study was 6.7 months, with a local remission rate of 73 %. Prognostic factors include a total dose of 15 Gy with a KPS score of 80%. None of the patients showed grade III or grade IV effects [29].

EBRT: Re-RT represents a challenge because adequate dose-fractionation and duration of RT to achieve speciﬁc goals (curative or palliative) is still not well known, and there is limited data available to establish its efﬁcacy. In a large cohort study by Brooks, 912 patients received definitive SABR (50 Gy in four fractions or 70 Gy in 10 fractions), 102 patients who developed isolated recurrence (49 with local recurrences and 53 with regional recurrences ) received salvage treatment in the form of re-SABR, and they concluded that patients who received salvage treatment had significantly improved survival (p < 0.006) [30].

PBT: The role of PBT has been explored by McAvoy et al. in which 102 patients from MD Anderson Cancer Center, Houston, Texas, underwent re-RT for intrathoracic recurrence for non-small cell lung cancer with a median re-RT dose of 60.48 EQD2 Gy; median overall survival observed was 14.71 months; distant metastasis-free survival was 11.43 months; whereas the addition of concurrent CT and higher EQD2 was found to be associated with improved OS [31].

Carbon ion: A single institutional study by Miyamoto et al. from the School of Medicine, Chiba University, Chiba, Japan, regarding carbon ion RT for stage 1 included 81 patients with cumulative dose from 59.4 to 95.4 GyE, showed optimal safety and efficacy with carbon ion therapy [32].

Esophageal Cancer

Neoadjuvant CTRT is the treatment of choice for mid- to low-thoracic esophageal cancers, followed by surgery. In contrast, definitive chemoradiation is the standard of care for locally advanced and cervical esophageal cancers. A report from Lancet Oncology by Pennathur et al. showed an overall five-year survival of patients with esophageal carcinoma ranging from 15% to 25%, in which 40%-60% had a recurrence. Various modalities have been tried in recurrent esophageal carcinomas, including external beam RT with IMRT and SABR and hyperthermia [33]. A study by Yamaguchi from the Department of Cancer Therapy Centre, Japan, with 31 patients, including 27 patients treated with concomitant CT and 14 receiving hyperthermia during re-RT, showed that patients treated with curative intent EBRT showed an objective response rate with acceptable toxicities. In contrast, the role of symptom palliation is restricted, especially with advanced primary [34].

EBRT with concurrent CT: RT mainly focuses on local irradiation of tumor tissue, while CT acts systemically. A synergistic effect of CTRT enhances the original efficacy with enhanced tumor cell kill and individual toxicities by each modality. A meta-analysis for 956 recurrent esophageal cancers treated either with EBRT alone (n = 476) and/or in combination with concurrent CT (n = 480) showed that the effective rates of RT alone and RT and CT were 45% and 85.7%, respectively, and good survival benefits with respect to the addition of single drug CT [35].

Brachytherapy: The role of endoluminal brachytherapy has been evaluated by Nicolay et al., in which 18 patients received brachytherapy either as a boost treatment for definitive or salvage treatment for recurrent cancer, and it showed a complete endoscopic response in follow-up procedures. They concluded that intraluminal brachytherapy could be applied, leading to good functional outcomes with less severe toxicities [36].

SABR: A single institutional study for the feasibility of SBRT by Seyedin et al., in which the median dose received was 27.5 Gy in five fractions, showed the feasibility of the SBRT with minimum treatment related to toxicities and tumor control [37].

Breast Cancer

Breast re-RT after breast conservation surgery (BCS) is increasingly used as salvage therapy with recurrent breast cancer. However, limited data is present on the safety and efficacy of various techniques of radiation in the re-RT scenario. Accelerated partial breast irradiation (APBI), hypofractionated EBRT, and brachytherapy are available options.

Brachytherapy: Multicatheter interstitial brachytherapy (MIB) in which catheters are placed in the breast around the lumpectomy cavity and radiation can be delivered directly to the tumor bed using HDR and LDR. The largest study by the Groupe Européen de Curiethérapie (GEC) and the ESTRO breast group by Hannoun-Levi et al. including 217 patients received MIB and reported a second local recurrence rate of 7% at 10-year follow-up [38].

Intraoperative RT (IORT): IORT emerged as an alternative technique with the benefit of performing salvage lumpectomy and re-RT at the same time. It uses an intra-beam system (50 kV photon beam mobile X-ray unit) with 20 Gy prescription to the applicator surface. Boehm et al. reported a single institutional retrospective analysis of 57 patients who received second breast conservative therapy with IORT, with a median follow-up of 24.5 months showing locoregional control of 89% and DFS with 81% concluding IORT as a tolerable treatment modality with acceptable toxicity in locally recurrent breast cancer avoiding mastectomy [39].

EBRT: Though second mastectomy is the standard treatment option post local recurrence following lumpectomy, surgery followed by re-RT is a reasonable option to avoid further recurrences. A study including 83 patients with breast cancer recurrence treated with re-RT with a dose of 45 Gy with 1.8 Gy per fraction by Janssen et al. resulted in excellent LC with favorable risk factors including younger age ( p < 0.05), lower tumor size ( p = 0.019), and N0 status (p = 0.005) [40]. In a different study that evaluated the role of re-RT with hypofractionation, Chen et al. adopted a regimen of 45 Gy in 30 fractions in 1.5 Gy per fraction in 34 patients from a landmark phase II trial RTOG 1014. They showed the effectiveness and tolerability of the regimen with acceptable toxicities [41].

Proton beam therapy (PBT): Proton therapy is superior to photon beam therapy in reducing doses of OARs, especially in the heart. A multi‐institutional analysis on proton beam therapy re-RT for breast cancer by Thorpe et al. showed efficacy and outcomes in PBT re-RT, in which 50 patients were included with median re-RT dose of 55.1 Gy to primary and/or regional lymph nodes, and showed acceptable toxicities and favorable LC [42].

Prostate Cancer

Prostate cancer constitutes a higher incidence, with the second most common cancer among older men, and recent advancements in earlier diagnosis and treatments reflected in decreased morbidities and mortality. RT is a local therapy in definitive, adjuvant or palliative settings [43]. Even after that, local failure rates noted by Agarwal et al. from the Cancer of the Prostate Strategic Urological Research Endeavor (CaPSURE), including 5277 men, are as high as 63% (p < 0.003) [44]. Various re-RT technologies have been searched and utilized to serve the re-RT purpose; the most relevant studies regarding each treatment modality are listed below.

Brachytherapy: HDR brachytherapy is a safe and evidence-based treatment for recurrent prostate cancer as it delivers very high doses of radiation in a very short treatment time precisely and is more conformal than LDR. A study including 115 patients who underwent HDR brachytherapy for locally recurrent prostate cancer between December 1999 and August 2008 with a regimen of 10 Gy per fraction using three applications showed performance safety with acceptable biochemical control and toxicity [45].

EBRT: A longstanding principle in radiation oncology states that after definitive EBRT has been delivered, re-RT will exceed normal tissue tolerances, leading to concerns of futility or potentially serious toxicity. However, new imaging and RT platforms have been developed that could allow for safer re-RT. A systematic review and meta-analysis by Corkum et al., in which 19 studies representing 13 cohorts were included with a total of 428 patients, showed LC rates of 83.2% and biochemical relapse free survival of 59.3%, concluding that salvage re-RT using EBRT, particularly with SBRT, is an emerging technique to treat the isolated local failure of prostate cancers [46].

Rectal Cancer

Despite multimodality approaches with preoperative RT, CT, and surgery (total mesorectal excision (TME)) have led to a decrease in the incidence of local relapse in 75% of the cases. Local recurrence occurs within two years of treatment in the form of deteriorated health-related quality of life parameters such as pelvic pain, fistula, and bowel obstruction. An Italian Association of Radiation and Clinical Oncology for Gastrointestinal Tumors (AIRO) gastrointestinal tumor study group led by Mantello stated that the treatment options for recurrence include radical surgery with overall survival up to 60% in negative margins. Re-RT causes an increase in the tumor-free margins (R0 resection) and provides symptom palliation in unresectable tumors. The most common fractionation was 30-40 Gy with a daily dose/fraction of 1.8-2 Gy. A total of 90-100 EQD2 dose was delivered in 46% of patients with modern conformal techniques and daily IGRT protocols. This study concluded that when performed with advanced technology, re-RT results in the good management of locally recurrent rectal cancer [47].

PBT: For patient management without surgery, proton beams offer the possibility of dose escalation with radical intent. Potential advantages of proton therapy in radical irradiation in rectal cancers include targeted recurrent sites that are not anatomically accessible or otherwise not suitable for brachytherapy. A single institutional retrospective study by Koroulakis et al. analyzed 28 patients with rectal cancer with prior pelvic RT receiving pencil beam scanning proton therapy (PBS-PT). The median dose was 44.4 Gy, showed a low acute toxicity rate (10.7%) and acceptable late toxicity (14.2%), and supported PBS-PT as a good option for high-risk patient populations [48].

Interstitial brachytherapy (ISBT): Brachytherapy has the advantage of delivering LDR radiation, which allows continuous DNA repair of sublethal damage in the normal tissues, resulting in a wider therapeutic index and rapid dose fall-off. A study for LDR brachytherapy by Bishop et al. from MD Anderson Cancer Center, including 20 patients who received ISBT for locally recurrent anorectal cancers, suggested a durable tumor control and long-term survival along with minimum long-term morbidity [49].

Gynecological Cancers

Treatment of primary cervical cancer includes surgery, RT, and CTRT, with recurrence mainly in the primary site, i.e., pelvic recurrence. The most common site includes the cervix and vagina. With the development of technologies such as SBRT and IGRT, effective LC with minimum tissue toxicity can be achieved with extensive clinical and physics counseling and consideration of patient prognostic factors such as the site of recurrence, dose, fractionation schedule, survival status, and DFS [50]. Various techniques for re-RT are mentioned below.

HDR ISBT: Prisciandaro et al. discussed the advantages of HDR ISBT, including flexible and highly conformal dose distribution, shorter treatment time (5-6 days), delivery to large deeply involved cervical recurrence, and tumor extended to parametrium and lower vaginal wall [51]. Table 4 summarizes relevant studies on HDR ISBT and their outcomes.

**Table 4:** Studies showing good response in cervical recurrence after high dose rate interstitial brachytherapy EQD2: Equivalent dose in 2 Gy fractions

Sr. No.	Study	Author Name	Dose Delivered	Outcome
1	Reirradiation using high-dose-rate interstitial brachytherapy for locally recurrent cervical cancer: a single institutional experience	Mabuchi et al. [52]	42 Gy/seven fraction	76.9% response rate
2	Use of interstitial brachytherapy in pelvic recurrence of cervical carcinoma: Clinical response, survival, and toxicity	da Silva et. al. [53]	40-60 Gy in 4-6 fraction	Control rate 67%
3	Reirradiation using high-dose-rate brachytherapy in recurrent carcinoma of uterine cervix	Mahantshetty et al. [54]	EQD2 42 Gy (37-46 Gy)	Two-year local control 44%

Permanent radioactive seed implantation (PRSI): The PRSI aims to place a microscopic radioactive source directly into or around the tumor to kill tumor cells by continuously emitting radiation from radionuclides. Radionuclides such as iodine 125 (I-125), palladium-103, cesium-131, and gold-198 are extensively utilized. Efficacy of image-guided radioactive I-125 seed (IGRIS) implantation for pelvic recurrent cervical cancer (PRCC) after EBRT has been evaluated by Qu et al. from the Department of Radiation Oncology, Peking University, China, in which 36 patients with PRCC received IGRIS. They concluded that IGRIS implantation could be a safe and effective salvage treatment for PRCC after EBRT, which could markedly release the pain [55]. Recurrence site, tumor volume, and dose were the main factors affecting efficacy.

SBRT: SBRT has been tried in many studies for inoperable small pelvic wall lesions and pelvic and para-aortic nodal recurrences. Seo et al., in their study of salvage stereotactic body RT for locally recurrent cervical cancer at the pelvic sidewall, included 23 patients with median SBRT dose ranging from 27 to 45 Gy (median 39 Gy) in three fractions and the fractional SBRT dose ranging from 9 to 15 Gy (median 13 Gy). They concluded that patients with tumor volume less than 30 cc have significantly longer two-year OS and local progression-free survival (PFS) than larger tumors (p < 0.001) [56]. A Korean radiation group (KROG 14-11) study evaluated LC and patient overall survival with SBRT using CyberKnife, including 71 patients and showed a two-year local PFS of 82.5%. They found out that SBRT for recurrent cervical cancer resulted in excellent LC, longer DFS, and higher biological effective dose (BED) treatments and can be considered therapeutic options for these patients [57].

Skin Cancer

RT has a pivotal role in the management of cutaneous malignancy. Important factors to be considered for the delineation of clinical fields and assessment of the clinical degree of late RT-induced changes include previous RT field and overlap with current field, dose fractionation schedule prescribed, and the presence of late RT infield changes such as telangiectasia and hypopigmentation. Re-RT should also be considered in the adjuvant setting in case of a patient undergoing surgery for reirradiated tissue.

Brachytherapy: A small study by Gauden from Launceston General Hospital, Tasmania, Australia, including 200 patients in which 74 cases received brachytherapy with a dose of 36 Gy in two weeks (3 Gy per fraction), showed 98% LC with 88% cosmesis rate [58].

## Conclusions

RT planning is advanced, yet many patients still experience local and regional failures, often associated with several prognostic factors, including tumor type, size, operability, lymph node involvement, and distant spread. Despite the lack of universal guidelines for re-RT, institutions follow their protocols, as low as reasonably achievable (ALARA) principles, or experiential judgment, emphasizing the critical role of clinical medical physicists. These professionals manage re-RT, considering aspects like medical records review, $\frac{\alpha}{\beta}$ ratio assumptions, organ repair percentage, cumulative EQD2 calculations, and safe biological dose estimations. Clinical medical physicists are key from decision-making to treatment and follow-up, necessitating a defined protocol for re-RT. Patient anxiety is also reduced with physicist counseling before treatment. Re-RT techniques include IMRT, SBRT, proton therapy, and brachytherapy, each offering different advantages and challenges, with IMRT commonly used for deep-seated, inoperable, and recurrent lesions. Effective reirradiation requires a nuanced understanding of tumor radiobiology for target contouring by oncologists and precise treatment planning by physicists, considering previous doses, recurrence timing, and current disease conditions.
